# Machine learning and predictive models: 2 years of Sars-CoV-2 pandemic in a single-center retrospective analysis

**DOI:** 10.1186/s44158-022-00071-6

**Published:** 2022-10-14

**Authors:** Michela Rauseo, Marco Perrini, Crescenzio Gallo, Lucia Mirabella, Karim Mariano, Giuseppe Ferrara, Filomena Santoro, Livio Tullo, Daniela La Bella, Paolo Vetuschi, Gilda Cinnella

**Affiliations:** 1 Department of Anesthesia and Intensive Care Medicine, University Hospital “Policlinico Riuniti di Foggia”, University of Foggia, Viale Pinto, 1, 71122 Foggia, Italy; 2 Department of Clinical and Experimental Medicine “InfoLab” Bioinformatics Facility Head, University Hospital “Policlinico Riuniti”, Viale Pinto 1, 71122 Foggia, Italy

**Keywords:** COVID-19, Intensive care unit, Machine learning, Predictive models, Non-invasive ventilation, Mechanical ventilation, MEWS, NEWS, Emergency department, Length of stay, Acute respiratory failure

## Abstract

**Background:**

Since January 2020, coronavirus disease 19 (COVID-19) has rapidly spread all over the world. An early assessment of illness severity is crucial for the stratification of patients in order to address them to the right intensity path of care.

We performed an analysis on a large cohort of COVID-19 patients (*n*=581) hospitalized between March 2020 and May 2021 in our intensive care unit (ICU) at Policlinico Riuniti di Foggia hospital.

Through an integration of the scores, demographic data, clinical history, laboratory findings, respiratory parameters, a correlation analysis, and the use of machine learning our study aimed to develop a model to predict the main outcome.

**Methods:**

We deemed eligible for analysis all adult patients (age >18 years old) admitted to our department. We excluded all the patients with an ICU length of stay inferior to 24 h and the ones that declined to participate in our data collection. We collected demographic data, medical history, D-dimers, NEWS2, and MEWS scores on ICU admission and on ED admission, PaO_2_/FiO_2_ ratio on ICU admission, and the respiratory support modalities before the orotracheal intubation and the intubation timing (early vs late with a 48-h hospital length of stay cutoff). We further collected the ICU and hospital lengths of stay expressed in days of hospitalization, hospital location (high dependency unit, HDU, ED), and length of stay before and after ICU admission; the in-hospital mortality; and the in-ICU mortality. We performed univariate, bivariate, and multivariate statistical analyses.

**Results:**

SARS-CoV-2 mortality was positively correlated to age, length of stay in HDU, MEWS, and NEWS2 on ICU admission, D-dimer value on ICU admission, early orotracheal intubation, and late orotracheal intubation. We found a negative correlation between the PaO_2_/FiO_2_ ratio on ICU admission and NIV. No significant correlations with sex, obesity, arterial hypertension, chronic obstructive pulmonary disease, chronic kidney disease, cardiovascular disease, diabetes mellitus, dyslipidemia, and neither MEWS nor NEWS on ED admission were observed.

Considering all the pre-ICU variables, none of the machine learning algorithms performed well in developing a prediction model accurate enough to predict the outcome although a secondary multivariate analysis focused on the ventilation modalities and the main outcome confirmed how the choice of the right ventilatory support with the right timing is crucial.

**Conclusion:**

In our cohort of COVID patients, the choice of the right ventilatory support at the right time has been crucial, severity scores, and clinical judgment gave support in identifying patients at risk of developing a severe disease, comorbidities showed a lower weight than expected considering the main outcome, and machine learning method integration could be a fundamental statistical tool in the comprehensive evaluation of such complex diseases.

## Introduction

Two years after the beginning of the coronavirus (COVID-19) pandemic, thanks to the positive results of vaccinations, we are slowly coming back to our lives and the mortality due to SARS-CoV-2 has dropped down, but the high morbidity and all the chronic associated consequences are still a concern. To date, more than 520 million confirmed cases have been reported, including more than 6.2 million deaths (https://covid19.who.int/).

Since the first SARS-Cov-2 case appeared in Italy, on 20 February 2020, almost 2 months after the first registered case in Wuhan (China), it has been clear the dimension of the challenge that our healthcare system had to face, one of the harder and bigger crises of our times.

The South of Italy, in the beginning, had the time to organize its emergency plan according to the North of Italy’s experience feedback but the unpredictability of the timing, evolution, and weight of the following waves put serious difficulties on our already fragile facilities.

From the beginning of the pandemic until 31 May 2021, we registered 250,419 confirmed SARS Cov-2 cases in Apulia, of whom 44763 in Foggia’s area (https://www.regione.puglia.it/web/speciale-coronavirus/elenco-bollettini-covid); the most affected area of Apulia considering the number of cases/100,000 inhabitants’ ratio.

In this crisis, many efforts have been spent in research to find simple and effective tools to identify deteriorating patients early, in order to optimize the path, therapy, and degree of intensification of care according to the clinical presentation.

For this purpose, the use of risk scores based on physiological parameters, has been clearly considered the right strategy to overcome this issue.

Many validated scores already used in intensive care clinical practice such as New Early Warning Score 2 (NEWS2) and Modified Early Warning Score (MEWS) have been used in retrospective cohorts of COVID-19 patients, with promising results, also in terms of survival prediction [1–4].

Furthermore, many efforts have been spent elaborating all the available data through artificial intelligence methods. These methods are gradually taking their place in healthcare as a high-performance technology helping the clinician in the identification of the fatality risk of a given patient with particular features, in the diagnosis process, or the prediction of disease spreading dynamics [5–9].

During the course of the current pandemic, machine learning has been used to develop different scores and algorithms [10–14] that help to identify, at an early stage, patients who are likely to develop a disease needing oxygen support, rehabilitation, hospitalization, or care intensification. These approaches make predictions relying on basic patient information and clinical symptoms, as well as travel history and discharge time of hospitalized patients.

Machine learning is a branch of the artificial intelligence field, which aims to provide computers with a learning capacity and well-defined algorithms to improve performance or make accurate predictions. These algorithms learn from past information available, introduced in the form of labeled training sets. Through the use of these labeled training sets, the supervised learning algorithms optimize the parameters of a statistical model so that a loss function is minimized. As a result, the trained model is then able to make predictions using input data that have never been used in the training phase. In order to ensure the adequate performance of the algorithm, the quality and size of the datasets used are fundamental.

We present an analysis of a large cohort of COVID-19 patients (*n*=581) hospitalized between March 2020 and May 2021 in our intensive care unit (ICU) at Policlinico Riuniti di Foggia hospital.

Through an integration of the scores, demographic data, clinical history, laboratory findings, respiratory parameters, correlation analysis, and the use of machine learning, our study aimed to (a) evaluate the efficacy and criticalities in managing patients needing an intensive care hospitalization due to SARS CoV-2 acute respiratory distress syndrome (C-ARDS) in terms of in-ICU, in-hospital mortality and length of stay according to the initial clinical presentation, (b) evaluate the application of clinical scores such as NEWS2 or MEWS on ED and ICU admission as a triage tool in order to address the patients in the right intensity of care path, and (c) search for variables of interest through multivariate analysis integrating them into machine learning algorithms to develop a model to predict the main outcome.

## Methods

This retrospective study was conducted at the Policlinico Riuniti di Foggia hospital, Italy. The developed dataset included the records of all adult patients with confirmed SARS-CoV-2 infection admitted into our ICU department between March 2020 and May 2021. The study was reviewed and approved by the local ethical committee and conformed to the principles outlined in the Declaration of Helsinki. All methods were performed in accordance with the relevant guidelines and regulations. Patient data were anonymized. Laboratory confirmation of SARS-CoV-2 was defined as a positive result of real-time reverse transcriptase–polymerase chain reaction assay of nasopharyngeal swabs.

We collected through a non-uniform sampling method the following variables: demographic data (age, gender), medical history (obesity, arterial hypertension, chronic obstructive pulmonary disease, chronic kidney disease, cardiovascular disease, diabetes mellitus), D-dimers, and NEWS2 and MEWS scores on ICU admission and on ED admission. We collected the PaO_2_/FiO_2_ ratio on ICU admission, the respiratory support modalities before the orotracheal intubation, and the intubation timing (early vs late with a 48-h hospital length of stay cutoff). We further collected the ICU and hospital lengths of stay expressed in days of hospitalization, hospital location (high dependency unit, HDU, ED), and length of stay before and after ICU admission; the in-hospital mortality; and the in-ICU mortality.

### Inclusion and exclusion criteria

We deemed eligible for analysis all adult patients (age>18 years) admitted to our department. We excluded all the patients with an ICU length of stay inferior to 24 h and the ones that declined to participate in our data collection.

### Statistical analysis

For the statistical univariate and bivariate analysis, we used the open-source software *Jamov*i [15]*.* The Kolmogorov–Smirnov test was used to examine distribution normality. The mean and standard deviations were used to describe normal continuous variables. The median and interquartile ranges were used to describe non-normal continuous variables. Categorical data were expressed as the frequency in percent.

For continuous variables as well as ordinal variables Spearman’s rank-order correlation was used in order to evaluate the type and strength of the relationship between variables. Spearman’s correlation coefficient was considered statistically significant when *p*<0.05. Two populations of the sample (patients admitted to HDU and patients not admitted to HDU) were compared by using the Mann-Whitney *U* test for continuous variables; contingency tables composed of nominal variables were evaluated with a chi-squared distribution.

### Machine learning methods integration

Based on both statistical correlations’ strength and clinical judgments, the variables of interest were evaluated by means of a multivariate analysis provided by Orange [15], an open-source machine learning software, whose algorithms are able to assign a predictive value to variables taken into consideration.

Multivariate analysis was performed by scoring variables through the Gini index. We selected the following variables among all the scored variables according to clinical observation and judgment for a multivariate graphic descriptive analysis: age, D-dimers on ICU admission, PaO_2_/FiO_2_ ratio on ICU admission, NIV, early OTI, late OTI, MEWS, and NEWS 2 on both ED and ICU admission, length of stay in HDU, diabetes mellitus, and chronic kidney disease. Based on the selected variables, a set of machine learning methods was developed in order to create a predictive model for the main outcome (i.e., in-ICU mortality). We evaluated ten supervised machine learning models for this prediction task: CN2 rule inducer, tree, kNN, random forest, Naïve Bayes, SVM, gradient boosting, SGD, logistic regression, and neuronal network.

A 10-fold stratified cross-validation test was used for an internal validation of the developed model. We used receiver operating characteristic (ROC) curves to compare the sensitivity and specificity of scores generated with different machine learning techniques.

## Results

### Cohort description

We collected data from a total of 581 consecutive patients with confirmed SARS-CoV-2 infection admitted into our ICU from March 2020 to May 2021. Among them, 28 patients were definitely excluded as they did not meet the inclusion criteria.

### Consort flow diagram

**Fig. 1:**
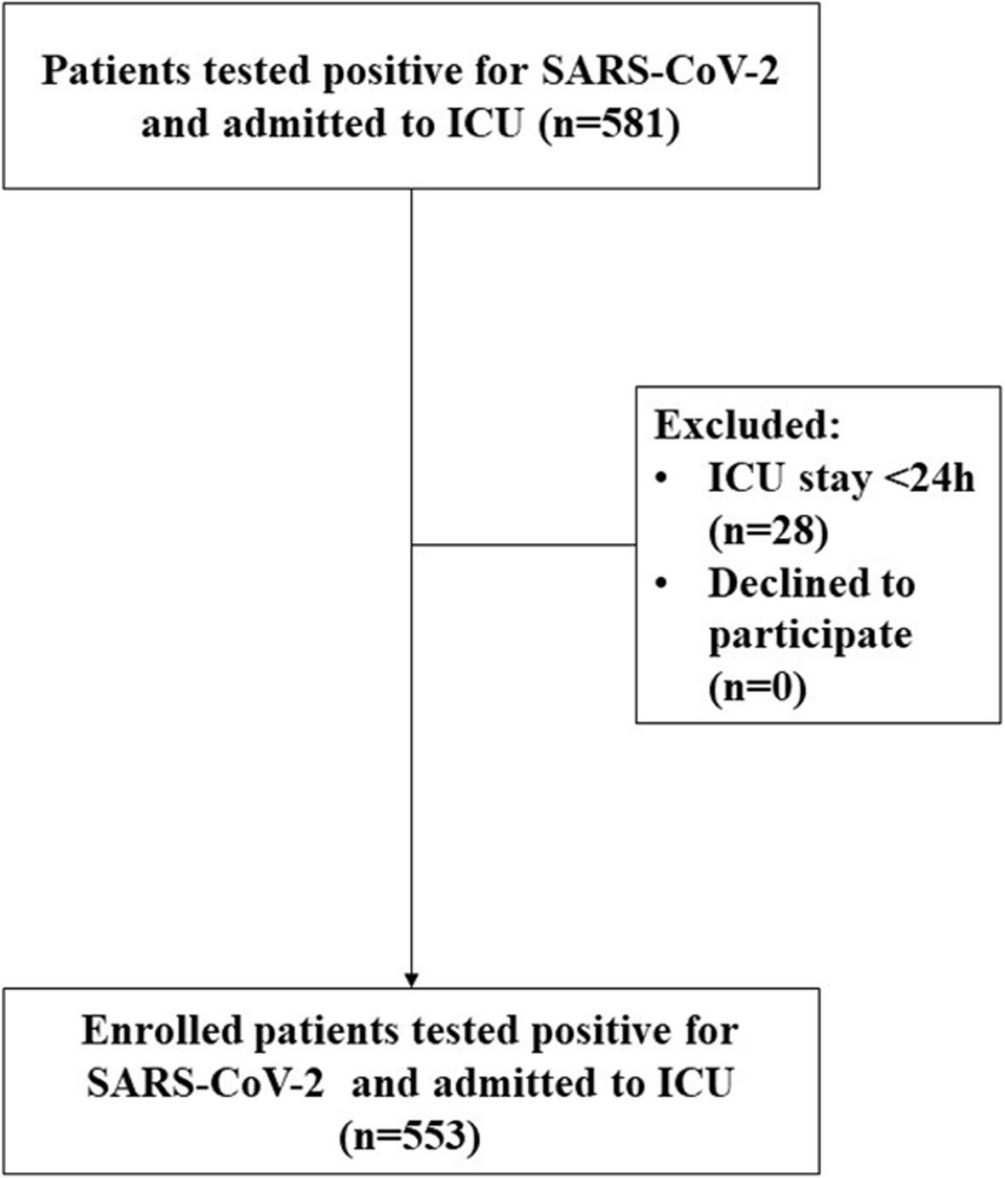
CONSORT flow diagram summarizing patients eligible for the study

553 patients were included at the end. The baseline demographic and clinical characteristics of the study population are presented in Table 1. 326 patients were admitted into our ICU after an ED stay, and 227 patients came to our department after a progressive worsening of their clinical conditions during their stay in medical wards or HDUs. More than two thirds of the admitted patients (372 out of 553, 67.2%) ranged from 60 to 79 years of age (Fig. 1).

**Table 1:**
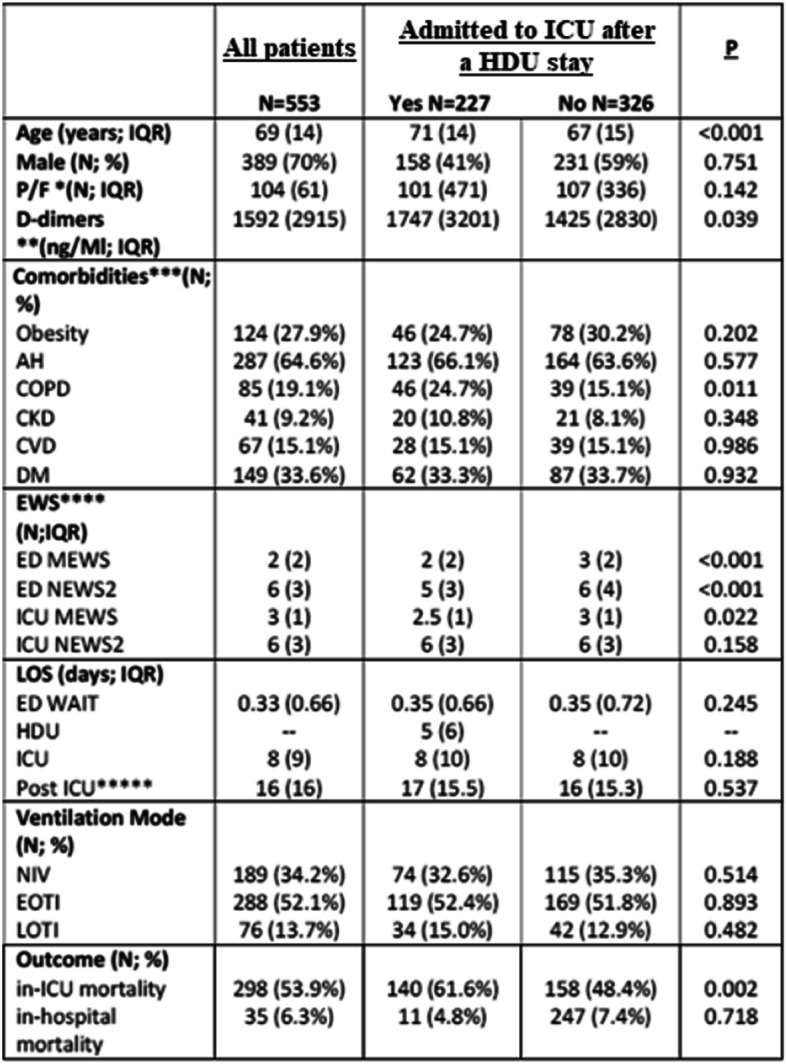
Data are presented as median (IQR) or number (percentage)

*Abbreviations: AH* arterial hypertension, *COPD* chronic obstructive pulmonary disease, *CKD* chronic kidney disease, *CVD* cardiovascular

Disease: *DM* diabetes mellitus, *EWS* Early Warning Score, *ED* emergency department, *ICU* intensive care unit, *MEWS* Modified Early Warning Score, *NEWS2* National Early Warning Score, *LOS* length of stay, *HDU* high dependency unit, *NIV* non-invasive ventilation, *EOTI* early orotracheal intubation, *LOTI* late orotracheal intubation, *H* hospital

*P/F ratio was evaluated for 478 patients (86.4%) of which were admitted to ICU after a HDU stay: Yes 202 (42.3%), No 276 (57.7%)

**D-dimers were evaluated for 431 patients (77.9%) of which were admitted to ICU after a HDU stay: Yes 170 (39.4%), No 261 (60.6%)

***Comorbidities were calculated on 444 patients (80%) of which were admitted to ICU after a HDU stay: Yes 186 (41%), No 258 (59%)

****EWS were calculated on patients with available information on that variable

*****Post-ICU LOS was measured on patients who survived in-ICU, 255 (46.1%) of which were admitted to ICU after a HDU stay: Yes 87 (38.3%), No 168 (51.6%)

### ED admission—MEWS and NEWS2

Upon admission to the emergency room, we collected MEWS of 347 out of 553 patients (62.7%), the overall median value of the MEWS was 2 (IQR 2; range 10). 312 out of 347 patients (89.9%) had a MEWS of less than 5 (range: 0–4). 35 out of 347 patients (10.1%) had a MEWS greater than or equal to 5 (range: 5–10). The median MEWS in patients admitted to ICU was 3 (IQR 2; range 10), while the median MEWS value of patients admitted to HDU was 2 (IQR 2; range 10).

Upon entering the ED, the NEWS of 314 out of 553 patients (56.8%) was calculated. The overall median value of NEWS2 was 6 (IQR 3; range 15). 200 of 314 patients (63.7%) had a NEWS2 of less than 7 (range: 0–6).

114 of 314 patients (36.7%) presented a NEWS2 greater than or equal to 7 (range: 7–15). The median NEWS2 in patients who were admitted to ICU was 6 (IQR 4; range 15), while the median NEWS2 value of patients admitted to an HDU was 5 (IQR 3; range 11).

The median time spent in the ED by 79% of patients (458 out of 553) was 10.5 h (IQR 16.5; range 302 h).

### ICU admission—MEWS and NEWS2

Upon arrival in ICU, we collected MEWS of 308 patients out of 553 (55.7%), whose overall median value was 3 (IQR 1; range 10). 278 out of 308 patients (90.3%) had a MEWS lower than 5 (range: 0–4).

Among these patients, 180 (64.7%) were transferred from the ED and 98 (35.3%) from an HDU. Thirty out of 308 patients (9.7%) were admitted with a MEWS greater than or equal to 5 (range: 5–9), 18 of them (60%) were transferred from the ED, and 12 (40%) from an HDU. The NEWS2 of 302 out of 553 patients (54.6%) was calculated. The overall median value was 6 (IQR 3; range 15).

160 out of 302 patients (53.0%) had a NEWS2 lower than 7 (range: 1–6) on their arrival in ICU. 97 patients (60.6%) were transferred from the ED, while 63 (39.4%) from an HDU. 142 out of 302 patients (47.0%) had a NEWS2 greater than or equal to 7 (range: 7–16) and 95 of them (66.9%) came from the ED, while 47 (33.1%) were from an HDU.

### PaO_2_/FiO_2_ ratio

We further evaluated the PaO_2_/FiO_2_ ratio of 478 out of 553 patients (86.4%) on ICU admission. 206 out of 478 patients (43.1%) had a PaO_2_/FiO_2_ ratio<100. 214 out of 478 patients (44.8%) had a P/F ratio 100< PaO_2_/FiO_2_ <200. 58 out of 478 patients (12.1%) had a PaO_2_/FiO_2_ ratio > 200.

### Ventilatory support

189 patients (34.2%) were exclusively treated with NIV, and 288 patients (52.1%) were connected to invasive mechanical ventilation support in the first 48 h after a failed NIV trial or due to several conditions on presentation, whereas 76 patients (13.7%) were connected after at least 48 h after a poor NIV clinical response.

Among the 189 patients exclusively treated with NIV, 164 (86.8%) survived, while 20 (10.6%) died in ICU and 5 (2.6%) died after transfer from ICU to rehabilitation wards. The overall mortality in this group of patients was 13.2%.

Among the 288 patients intubated within 48 h (early intubation group), 51 survived (17.7%), 213 died in ICU (74.0%), and 24 (8.3%) died in other rehabilitation wards. The overall mortality in this group was 82.3%.

Among the 76 patients connected to invasive mechanical ventilation support after at least 48 h (delayed intubation group), 5 survived (6.5%), while 65 (85.5%) died in ICU and 6 (7.9%) died in another rehabilitation ward after ICU transfer; the overall mortality was 93.4%.

### Outcome

Overall mortality during the three waves (March 2020–May 2021) was 60.2% (deaths, *n*=333; patients, *n* =553). The ICU deaths and the deaths after transfer from ICU to other rehabilitation wards were 298 (53.9%) and 35 (6.3%), respectively. Death occurred in 60.4% of female patients (*n*=164) and 60.2% of male patients (*n*=389).

The median age of death was 72 years (IQR 1, range 56) for the ICU and 72 (IQR 13.5, range 38) for the patients who died after transfer from the ICU to another rehabilitation ward. 220 patients survived (39.8%), of which 29.5% were female (*n*=65) and 70.5% male (*n*=155).

### Bivariate analysis

Spearman’s rank correlation matrix showed in our sample how SARS-CoV-2 mortality was positively correlated to age (*ρ* = 0.404, *p* value <0.001), length of stay in HDU (*ρ* = 0.166, *p* value <0.001), MEWS (*ρ* = 0.222, *p* value<0.001), and NEWS2 on ICU admission (*ρ* = 0.257, *p* value <0.001), D-dimers value on ICU admission (*ρ* = 0.259, *p* value <0.001), early orotracheal intubation (*ρ* = 0.455, *p* value <0.001), and late orotracheal intubation (*ρ* = 0.269, *p* value <0.001). We found a negative correlation with the PaO_2_/FiO_2_ ratio on ICU admission (*ρ* = −0.267, *p* value <0.001) and NIV (*ρ* = −0.675, *p* value <0.001).

No significant correlations with sex, obesity, arterial hypertension, chronic obstructive pulmonary disease, chronic kidney disease, cardiovascular disease, diabetes mellitus, dyslipidemia, and neither MEWS nor NEWS on ED admission were observed.

### Multivariate analysis

For our multivariate analysis, we considered only the ICU mortality excluding the records related to the in-hospital mortality outcome (*n*=35) because of the low statistical contribution eventually apported to the machine learning algorithms thus reducing bias in our analysis. We performed our multivariate analysis on 518 patients’ records starting with a scoring of the variables through the GINI score (Table 2).

**Table 2:** Variables’ ranking results. Variables are listed from the most linked to the outcome to the least

Model	AUC	CA	F1	Precision	Recall
*Logistic regression*	0.914	0.857	0.856	0.859	0.857
*Neural network*	0.913	0.846	0.845	0.845	0.846
*Naïve Bayes*	0.909	0.857	0.855	0.860	0.857
*Random forest*	0.907	0.847	0.846	0.849	0.847
*Gradient boosting*	0.899	0.844	0.843	0.844	0.844
*SVM*	0.880	0.849	0.843	0.844	0.844
*CN2 rule inducer*	0.843	0.774	0.774	0.775	0.774
*SGD*	0.836	0.849	0.847	0.853	0.849
*Tree*	0.774	0.786	0.787	0.789	0.786
*kNN*	0.593	0.589	0.583	0.582	0.589

**Fig. 2:**
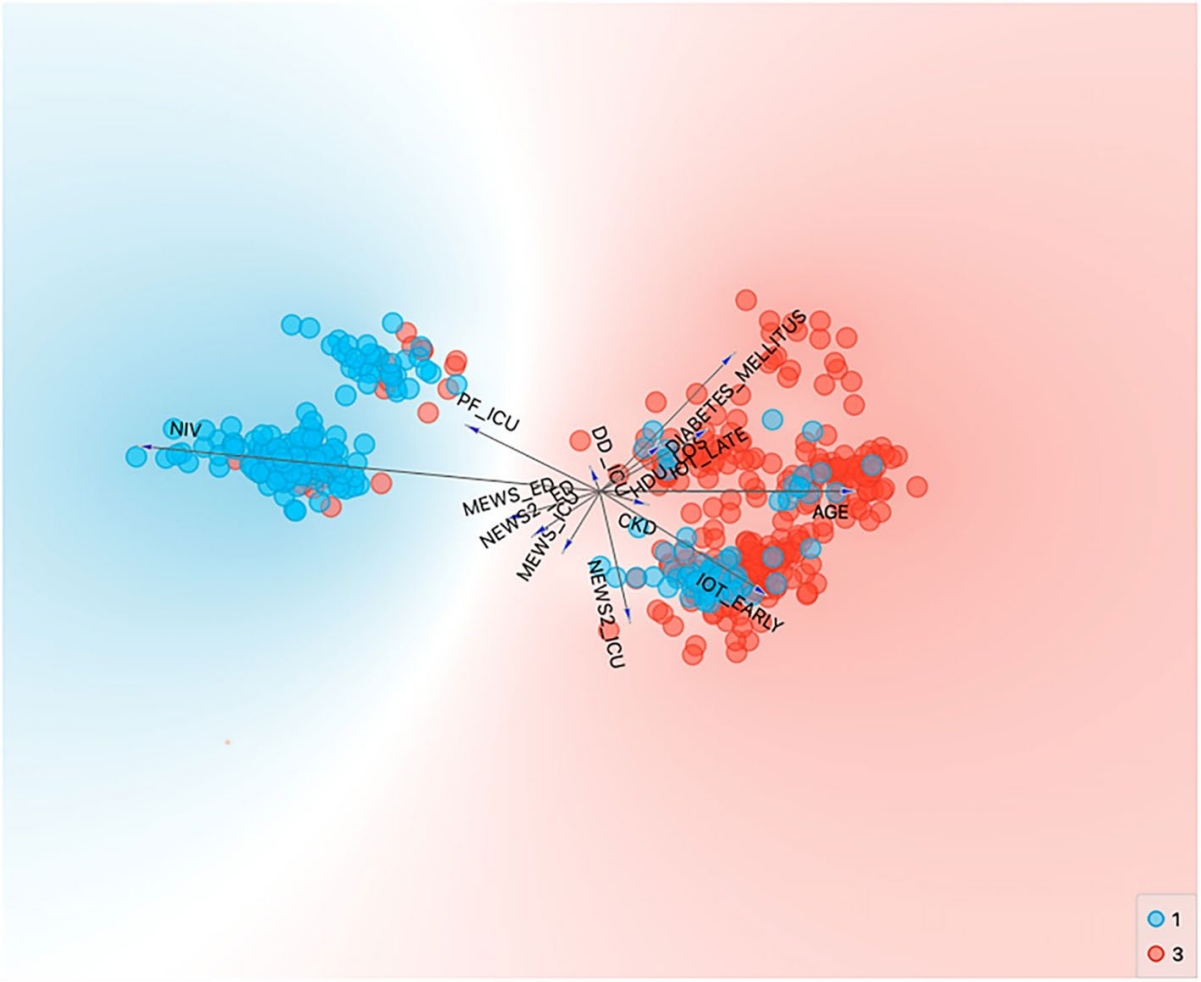
FreeViz graphic of the scored variables. The blue spots represent the survived patients, the red spots represent the dead patients

For the variable visualization, we used a FreeViz graphic (Fig. 2). FreeViz optimizes a linear projection and displays the projected data in a scatterplot. Through a gradient optimization approach, the target projection is found aiming to separate the instances of different classes in class-labeled data. Each data instance is here described with a set of features which are our selected predictive variables and labeled with a class according to the outcome.

In our qualitative analysis as shown in Table 2 and in Fig. 2, we could find five strongly associated with the outcome (in-ICU mortality) variables: age, PaO_2_/FiO_2_ ratio on ICU admission, NIV, early OTI, and late OTI.

Considering all the pre-ICU variables (age, PaO_2_/FiO_2_ ratio on ICU admission, NIV, early OTI, late OTI, MEWS, and NEWS on both ED and ICU admission, D-dimers on ICU admission, length of stay in HDU, diabetes mellitus, chronic kidney disease, obesity, arterial hypertension, dyslipidemia, COPD, ED stay in hours), none of the machine learning algorithms performed well in developing a prediction model enough accurate and statistically strong to predict the outcome.

A secondary multivariate analysis focused on the ventilation modalities and the main outcome confirmed the strong existing correlation as shown in Table 3.

**Table 3:** Results of the secondary multivariate analysis focused on the ventilation modalities and the main outcome. In the table, listed are all the machine learning-tested models and their performances from the most performing to the least

Model	AUC	CA	F1	Precision	Recall
*Logistic regression*	0.914	0.857	0.856	0.859	0.857
*Neural network*	0.913	0.846	0.845	0.845	0.846
*Naïve Bayes*	0.909	0.857	0.855	0.860	0.857
*Random forest*	0.907	0.847	0.846	0.849	0.847
*Gradient boosting*	0.899	0.844	0.843	0.844	0.844
*SVM*	0.880	0.849	0.843	0.844	0.844
*CN2 rule inducer*	0.843	0.774	0.774	0.775	0.774
*SGD*	0.836	0.849	0.847	0.853	0.849
*Tree*	0.774	0.786	0.787	0.789	0.786
*kNN*	0.593	0.589	0.583	0.582	0.589

**Fig. 3:**
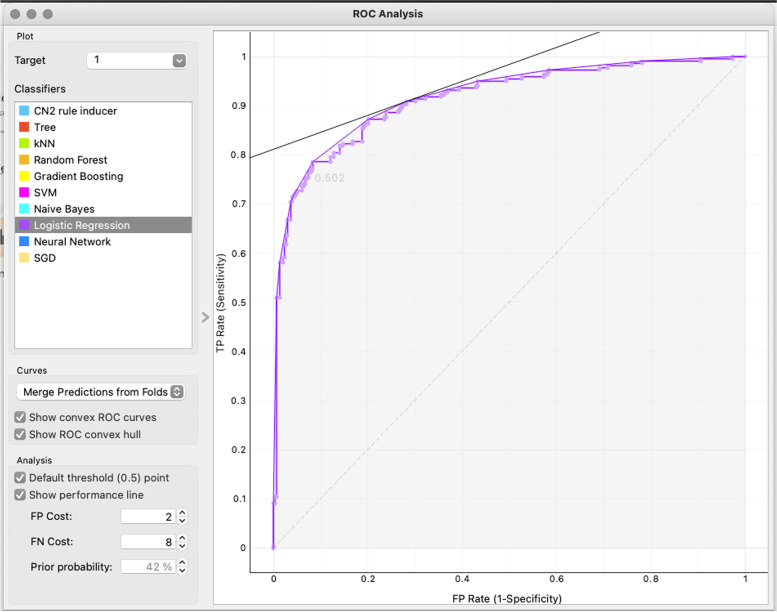
ROC curve of the logistic regression model in the secondary multivariate analysis

We used for the internal validation of the lo a 10-fold stratified cross-validation test obtaining the ROC curve in Fig. 3.

## Discussion

**Fig. 4:**
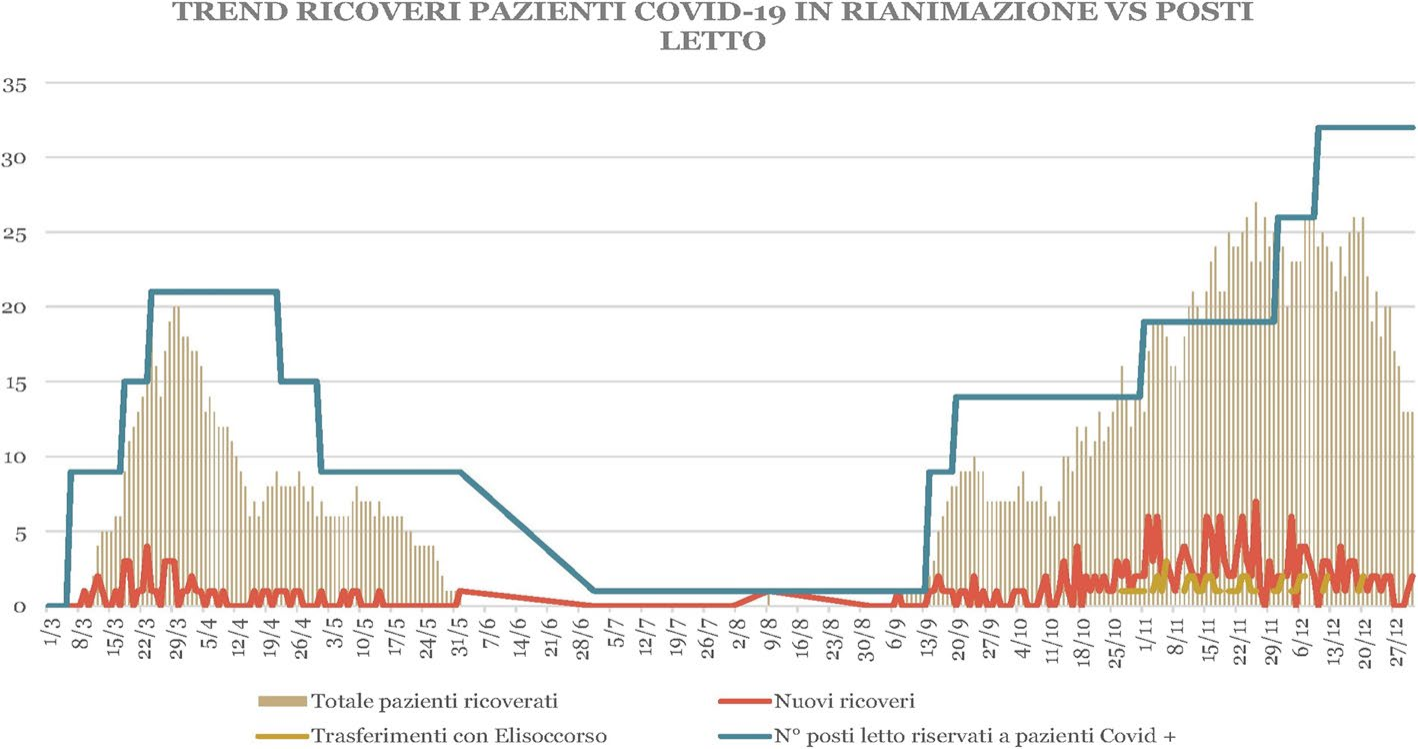
Trends of number of ICU patients vs beds availability in Foggia from January 03, 2020, until January 01, 2021

The main result and observation in our study pointed out the importance of the chosen ventilatory support, and patients exclusively treated with NIV showed a higher survival rate such as the patients in the early intubation group (Fig. 4).

We should surely consider these results by evaluating patients’ clinical conditions according to the chosen ventilatory support. The NIV group on ICU arrival had PaO_2_/FiO_2_ (median 130; IQR 65.5), MEWS (median 2; IQR 1), NEWS2 (median 6; IQR 2) while the early OTI group PaO_2_/FiO_2_ ratio (median 93; IQR 47), MEWS (median 3; IQR 1), and NEWS2 (median 8; IQR 3).

We could speculate that if NIV application before intubation is not significantly associated with an increased mortality, it may be clinically safer to use intubation as a last chance, as many authors suggested [16–20].

SARS-CoV-2 showed us the importance of logistics and the consequences connected to the difficulties in facing a huge healthcare challenge. The discrepancy between the availability of ICU and HDU beds and the number of patients needing treatment was the most evident logistical problem.

This condition led to prolonged waiting time in ED or even to temporary transfers to less equipped and skilled wards in order to treat patients’ clinical conditions. The delay most probably affected their outcome, as suggested by Spearman’s Rho of this variable (*ρ* = 0.166, *p* value <0.001) that showed a moderately statistically significant correlation.

The massive redeployment of medical and nursing staff, as previously mentioned, was fundamental to reach the objective of a structural reorganization but many of them were placed in a new, less known working situation, facing an unfamiliar disease, and in some cases operating with limited intensive care/emergency medical experience.

Finding simple and effective tools to identify deteriorating patients early, in order to optimize the path, therapy, and degree of intensification of care according to the clinical presentation was one of the objectives, and the use of risk scores has always been recognized as fundamental in this purpose in different kinds of diseases and also in Sars-Cov-2 patients.

We retrospectively evaluated the efficacy of MEWS and NEWS2 as triage tools to better address patients in the right intensity of care path.

We considered differences in stratification of the patients evaluating severity according to defined cutoffs in both of the scores. The two scores showed a statistically significant difference in assigning the proper degree of severity to the patients. On ED admission, MEWS scored 35 patients above the critical threshold, whereas NEWS2 assigned the higher severity score to more than 114 patients. On ICU admission, the two scores showed even greater differences, since the MEWS assigned higher scores to 30 patients, while 142 patients had a NEWS2 score over the critical threshold.

Spearman’s rank correlation matrix highlighted a medium value of correlation for both scores on ICU admission, with MEWS slightly lower (*ρ*= 0.222, *p* value <0.001) than NEWS2 (*ρ* = 0.257, *p* value <0.001), no significant correlation emerged for both scores at ED admission.

Reasonably, these differences lie in the absence of the SpO_2_ parameter in the MEWS, whereas this parameter is considered in the NEWS2.

We can conclude that especially high NEWS2 at hospital admission in the ED can predict ICU transfer and a higher risk of mortality, such as demonstrated by a little group of studies [1, 2]. As already shown in many publications, age represents a highly statistically significant variable (*ρ* = 0.404, *p* value <0.001) for the outcome [21, 22].

PaO_2_/FiO_2_ ratio as expected showed a moderate significant negative correlation with the in-ICU mortality outcome (*ρ* −0.267, *p* value <0.001) while D-dimers levels were positively correlated to the outcome (*ρ* = 0.259, *p* value <0.001), as known in literature [23–25].

Despite a large number of studies in which the mortality rate is higher in comorbid patients [21, 22, 26] in our retrospective study, comorbidities such as obesity, arterial hypertension, chronic obstructive pulmonary disease, and cardiovascular disease did not contribute to the worse outcome. Diabetes mellitus and chronic kidney disease showed a very low significant correlation (respectively, *ρ* = 0.143, *p* value = 0.003 and *ρ* = 0.109, *p* value = 0.022.

The application of machine learning algorithms to predict outcomes by developing a prediction model based on selected variables is a powerful tool increasingly used in biometrics and it was extensively used in COVID-19 [10, 11, 13, 27, 28].

We could not develop a well-performing predictive model, but this kind of tool would be able to give in a reasonable time, through different applications, according to the context, a pool of different advantages such as more effective triaging and optimization of health care system resources and personnel, decrease potential health care-associated infections, and improved clinical outcomes for the patients, especially during pandemics and complex situations from a logistical point of view. Multivariate visualization, as used in our analysis, is another tool used in biomedical data mining, and may provide a starting point in explorative analysis in the field of multivariate analysis.

In such complex diseases, analyzing data sets with many features, the principal problem to solve is which features to visualize and how to combine them in the visualization.

Obtaining a good visualization of the highest-contributing comorbidities, symptoms, and biochemical features at the same time helps clinicians to better understand, predict, and explain potential pathology clinical evolutions and prognoses.

### Limitations

In this study, we only included participants from a single hospital. It is possible that different confounding risk factors biased the outcome: patients’ socioeconomic status, nutrition conditions, and especially the accessibility of care considering the oversaturation of the healthcare system during the pandemic waves.

In the study period, we should also take into count the start of the vaccination campaign and the different therapeutic approaches that changed along the time. Furthermore, all the available complete data included only patients were evaluated at the time of admission to ED and ICU. We retained appropriate to share our findings to give our contribution to investigating whether different healthcare, logistic, or demographic backgrounds influenced critically ill patients’ outcomes.

## Conclusion

In conclusion, even if the multivariate analysis did not allow us to extrapolate a predictive model on the basis of the pre-ICU variables considered, machine learning methods are gradually helping evidence-based medicine improve, and modify the clinical, therapeutic, and logistical approach to such complex diseases. From our analysis, interesting correlations emerged, reinforcing fundamental concepts in the management of covid-related respiratory failure.

The choice of the right ventilatory support and the right timing are crucial, severity scores, and clinical judgment help in identifying patients at risk of developing a severe disease, comorbidities may have a lower weight than expected considering the outcome although further studies are certainly needed.

## Data Availability

The datasets during and/or analyzed during the current study available from the corresponding author on reasonable request.
